# Detailed characterization of tumor infiltrating lymphocytes in two distinct human solid malignancies show phenotypic similarities

**DOI:** 10.1186/s40425-014-0038-9

**Published:** 2014-11-18

**Authors:** Magdalena Kovacsovics-Bankowski, Lana Chisholm, Jonna Vercellini, Christopher G Tucker, Ryan Montler, Daniel Haley, Philippa Newell, Jun Ma, Paul Tseng, Ronald Wolf, John T Vetto, Chet Hammill, Paul Hansen, Andrew D Weinberg

**Affiliations:** Earle A. Chiles Research Institute, Providence Cancer Center, Portland Providence Medical Center, 4805 NE Glisan St, Portland, Oregon 97213 USA; Agonox Inc, 4805 NE Glisan St, Portland, Oregon 97213 USA; Providence Gynecologic Oncology, Providence Cancer Center, Portland Providence Medical Center, 4805 NE Glisan St, Portland, Oregon 97213 USA; OHSU, division of oncological surgery and OHSU Knight Cancer Center, 3303 SW Bond Ave, Portland, OR 97239 USA; Providence Surgical Oncology, Providence Cancer Center, Portland Providence Medical Center, 4805 NE Glisan St, Portland, Oregon 97213 USA

**Keywords:** Tumor infiltrating lymphocytes, Regulatory T cells

## Abstract

**Background:**

We examined the phenotype and function of lymphocytes collected from the peripheral blood (PBL) and tumor (TIL) of patients with two different solid malignancies: colorectal cancer liver metastases (CRLM) and ovarian cancer (OVC).

**Methods:**

Tumor and corresponding peripheral blood were collected from 16 CRLM and 22 OVC patients; immediately following resection they were processed and analyzed using a multi-color flow cytometry panel. Cytokine mRNA from purified PBL and TIL CD4^+^ T cells were also analyzed by qPCR.

**Results:**

Overall, we found similar changes in the phenotypic and cytokine profiles when the TIL were compared to PBL from patients with two different malignancies. The percentage of Treg (CD4^+^/CD25^+^/FoxP3^+^) in PBL and TIL was similar: 8.1% versus 10.2%, respectively in CRLM patients. However, the frequency of Treg in primary OVC TIL was higher than PBL: 19.2% versus 4.5% (p <0.0001). A subpopulation of Treg expressing HLA-DR was markedly increased in TIL compared to PBL in both tumor types, CRLM: 69.0% versus 31.7% (p = 0.0002) and OVC 74.6% versus 37.0% (p <0.0001), which suggested preferential Treg activation within the tumor. The cytokine mRNA profile showed that IL-6, a cytokine known for its immunosuppressive properties through STAT3 upregulation, was increased in TIL samples in patients with OVC and CRLM. Both TIL populations also contained a significantly higher proportion of activated CD8^+^ T cells (HLA-DR^+^/CD38^+^) compared to PBL (CRLM: 30.2% vs 7.7%, (p = 0.0012), OVC: 57.1% vs 12.0%, (p <0.0001)).

**Conclusion:**

This study demonstrates that multi-color flow cytometry of freshly digested tumor samples reveals phenotypic differences in TIL vs PBL T cell sub-populations. The TIL composition in primary and metastatic tumors from two distinct histologies were remarkably similar, showing a greater proportion of activated/suppressive Treg (HLA-DR^+^, CD39^+^, CTLA-4^+^ and Helios^+^) and activated cytotoxic T cells (CD8^+^/HLA-DR^+^/CD38^+^) when compared to PBL and an increase in IL-6 mRNA from CD4 TIL.

**Electronic supplementary material:**

The online version of this article (doi:10.1186/s40425-014-0038-9) contains supplementary material, which is available to authorized users.

## Background

Tumor immunotherapy has emerged as an important treatment modality for cancer patients. Historically, melanoma and renal cell carcinoma have been described as tumors that respond to immunotherapy. However, recent clinical trials have shown that a variety of other tumor types are also responding to immunotherapy [1-3].

The mechanism(s) of tumor rejection following immunotherapy treatment have been difficult to define. Until recently, most immunologic analyses in cancer patients have been conducted on PBL or tissue sections, where samples are more easily obtained. However, it is the composition of the immune cells at the tumor site that is likely to be most important; and the tumor infiltrating lymphocytes (TIL) may be quite different from the PBL. Furthermore it is extremely hard to perform immune subset analyses by immunohistochemistry. Therefore to gain a deeper understanding of how immunotherapy could potentially affect the tumor microenvironment, it would be important to first characterize the subset distribution and phenotype of the immune cells in progressively growing tumors.

The purpose of this study was to compare the phenotype and function of freshly isolated TIL to PBL in patients with progressively growing tumors by flow cytometry. Several studies, using primarily immunohistochemistry, have shown that greater frequencies of Treg cells within the tumor environment leads to a decrease in patient survival [4,5]. Increased Treg inhibitory function has been associated with increases in several surface and/or intra-cellular markers. Treg cells with the greatest suppressive capacity express high levels of HLA-DR and CD39 on their surface and a decline in this population has been associated with renal graft rejection or premature delivery [6,7]. In contrast to Treg frequency and phenotype, increased levels of CD8^+^ T cells in TIL has been associated with a positive clinical outcome in patients with CRC [4]. However, these studies did not examine the activation status of CD8^+^ T cells. We have used the co-expression of HLA-DR and CD38 as well as the proliferation marker Ki-67 to determine the activation status of CD8^+^ T cells isolated from TIL vs blood.

In this study, we analyzed fresh tumor and paired peripheral blood samples from patients with CRC and OVC; we compared the function and phenotype of lymphocytes from TIL to those in peripheral blood. The first group was composed of patients undergoing resection of colorectal cancer liver metastases (CRLM), while the second enrolled women undergoing cytoreductive surgery for ovarian cancer.

CRC is the third most common cause of cancer-related deaths worldwide and liver is the most common site of this metastatic disease. Fifteen percent of patients with CRC, are diagnosed with liver metastasis at the same time as their primary tumor and another 15% will develop liver metastases after resection of the primary tumor [8]. Surgical resection is the primary treatment option for operable metastases but is associated with a recurrence rate of 50-90% [9-11]. The medical treatment after surgical resection has traditionally been cytotoxic chemotherapy and recent studies have suggested that the immune response has important prognostic implications for CRC patients [5,12].

Ovarian Cancer represents only 3% of female cancers, but it is the fifth leading cause of cancer-related death in women. Most patients are diagnosed in advanced stage (spread beyond the pelvis, 75%) due to lack of effective early detection strategies. Surgical debulking is the standard of care followed by chemotherapy with paclitaxel and carboplatin. Unfortunately, the relapse rate after treatment is high, up to 75%. Many different strategies have been attempted to reduce relapse rates with little success. Several studies summarized in a meta-analysis by Hwang et al. [13], have suggested that the composition of TIL also has prognostic significance.

The results of this study demonstrate that the composition of T lymphocytes within CRLM and ovarian tumors is significantly different than peripheral blood T lymphocytes from the same patient. Within the tumor microenvironment, there was a significant increase in both activated Treg and activated CD8^+^ T cells. Moreover, we found that regardless of the tumor type, tumor location, or whether the tumor was a primary or metastatic deposit, the composition of lymphocytes within the tumors were very similar.

## Results

### Patients

From September 2009 to September 2012, under the approval of our institute’s Institutional Review Board (Providence Portland Medical Center, IRB), 16 patients with CRLM (Table 1) and 22 patients with OVC were enrolled in the study. Biopsy specimens and body fluids were received and processed as described in Methods. CRLM patient’s age ranged from 30 to 84 years with a median of 67 years. The male to female ratio was 1:1. The primary CRC tumor was resected between 10 to 51 months prior to the resection of CRLM, with a median of 20 months. OVC patients’ age ranged from 51 to 88 years with a median of 63.5 years. The majority of patients were diagnosed with serous carcinoma 17/22. Two were diagnosed with endrometroid adenocarcinoma, two with peritoneal carcinoma (1 serous and 1 Muellerian) and one with carcinosarcoma. None of the OVC patients were pre-treated prior to obtaining the tumor resections hence the OVC patients were not included in Table 1.

**Table 1:** **Patient’s characteristics**

**CRI number**	**Age at liver procedure**	**Sex**	**Primary tumor T stage**	**Primary tumor N stage**	**Primary tumor M stage**	**Post primary resection chemotx**	**Adjuvant chemo before liver resection**	**Time between resection and chemotx (Days)**
CRI 1010	60	F	3	0	0	None	Bevacizumab, Oxaliplatin, Capecitabine	49
CRI 1012	53	M	3	0	0	5FU + Leukovorin	FOLFOX + avastin	209
CRI 1091	60	M	3	2	1	FOLFOX	FOLFOX	52
CRI 1101	47	F	3	1	1	FOLFOX + avastin	FOLFOX + avastin	36
CRI 1109	70	M	4	1	1	XELOX	XELOX	52
CRI 1102	55	M	3	2	1	FOLFOX + avastin	FOLFOX + avastin	133
CRI 1126	60	F	3	1	1	FOLFOX	FOLFOX + avastin	487
CRI 1129	79	M				None	Capecitabine	53
CRI 1109	70	M	4	1	1	XELOX	XELOX	123
CRI 1208	83	F	3	1	0	XELOX	FOLFIRI + avastin	103
CRI 1109	70	M	4	1	1	n/a	n/a	n/a
CRI 1197	73	F			1	FOLFOX	FOLFOX	89
CRI 1400	77	M	3	0	1	Modified FOLFOX	None	185
CRI 1405	33	F	3	0	1	FOLFOX	FOLFOX	36
CRI 1404	50	F	3	0	0	None	FOLFOX	35
CRI 1513	29	F	4a	2b	0	FOLFOX	None	None

### T cell composition: PBL versus TIL

TIL isolation was successful for majority of tumor samples obtained. CRLM (n = 16) had fewer cells/gram of tissue compared to the OVC samples (n = 21) (median 1.5×10^6^ cells/g vs 2.7×10^6^ cells/g). The median percentages for CD3^+^ T cells after ficoll separation were 28.2% in primary OVCs, 53.1% within the omental metastases, 64.3% within ascites and 47.5% from PBMC (Tables 2 and 3). The percentage of CD3^+^ T cells from CRLM within the lymphocyte gate were, 35.8% for TIL and 53% for PBMC. The percentage of CD3^+^ T cells was calculated as a percentage of a standard FSC and SSC lymphocyte gate [14]. Within the CD3^+^ T cell population, CD4^+^ T cells represented 42.4% of the OVC primary tumor, 48.2% of the omental metastases and 49.8% of the CRLM TIL. Approximately 30% of the infiltrating lymphocytes in all tumor samples were CD8^+^ T cells, 30.8% for CRLM, 34.5% for primary OVC, 33.4% for omental metastases and 31.4% for ascites. The median CD4/CD8 ratio in the blood was very similar for both groups of patients (2.4 and 2.3). The median CD4/CD8 ratio was lower in T cells isolated from tumors (1.6 for CRLM, 1.3 for OVC primaries, 1.4 for omental mets and 1.3 for ascites). Detailed flow cytometric analyses of CD4^+^ and CD8^+^ T cells with other markers were performed as described in Additional file 1: Figure S1. This scheme was throughout the entire manuscript to calculate percentages in each group.

**Table 2:** **T cell distribution in CRLM**

**CRC liver metastasis**	**Median (25th-75th percentile) percentages**	
**Cells**	**Blood (n = 16)**	**TIL (n = 16)**
CD3+ T cells	53.0 (44.2-60.6)	35.8 (16.0-42.0)
CD4+ T cells	56.45 (51.4-73.6)	49.75 (32.1-53.9)
CD8 + T cells	21.15 (14.6-36.7)	30.75 (22.1-37.2)
CD4:CD8 ratio	2.4 (1.4-4.7)	1.6 (0.9-2.3)

**Table 3:** **T cell distribution in OVC tumor specimens**

**Ovarian**	**Median (25th-75th percentile) percentages**			
**Cells**	**Blood (n = 21)**	**Ascites (n = 16)**	**TIL (n = 21)**	**Met (n = 17)**
CD3+ T cells	47.5 (31.5-62.0)	64.30 (40.2-75.2)	28.2 (17.8- 52.3)	53.1 (25.1-62.3)
CD4+ T cells	62.3 (51.9-73.8)	49.0 (35.9-64.5)	42.4 (36.1-45.1)	48.2 (40.8-53.3)
CD8+ T cells	25.1 (18.7-36.6)	31.4 (25.8-48.5)	34.5 (22.6-47.5)	33.4 (23.1-39.0)
CD4:CD8 ratio	2.3 (1.4-4.0)	1.3 ( 0.8-2.5)	1.3 (0.8-1.8)	1.4 (1.1-2.0)

### Characterization of CD4^+^ T cell

**Figure 1:**
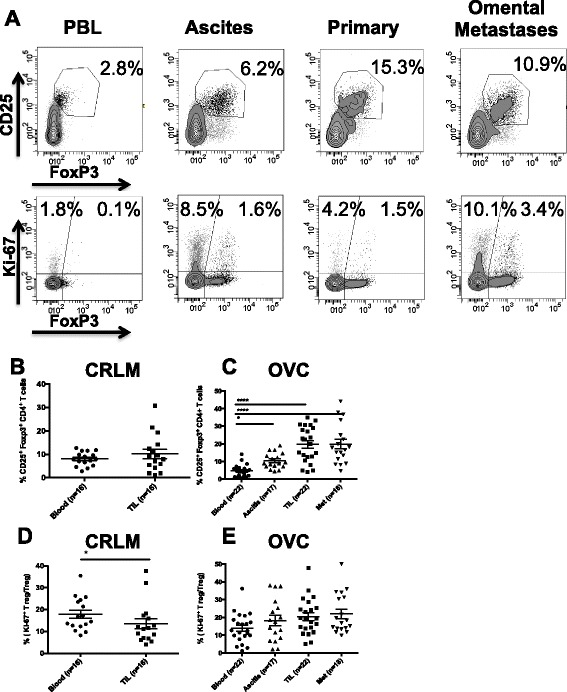
**CD4** ^**+**^ **T cell analysis. A**. CD3^+^ CD4^+^ T cells collected from PBL, ascites, primary tumor and omental metastases were analyzed for CD25 and FoxP3, upper panels and Ki-67 and Foxp3, lower panels. Percentage of CD25^+^ FoxP3^+^, Treg in PBL and TIL of CRC patients (n = 16) **B**, and in OVC patients (n = 22) **C**. Percentage of proliferating Treg in CRC samples **(D)** and in OVC samples **(E)**.

A representative dot plot analysis for CD4^+^ cells from an OVC sample is shown in Figure 1A. Using the gating scheme in Figure 1A we found that the mean percentage of CD4^+^ CD25^+^ FoxP3^+^ Treg within primary and omental mets in ovarian cancer patients were significantly increased compared to PBL (Figure 1C); however, in CRLM patients there was no significant difference in the mean percentage of Treg in TIL compared to blood (8.1% and 10.2% respectively, Figure 1B). We observed that the mean percentage of Treg in the peripheral blood was lower in OVC compared to CRC patients (4.5% vs 8.1%) (Figure 1B-C). However, the mean percentage of Treg within the primary or the omental OVC metastases was 4 times greater than the blood, reaching an average of 19.2% and 18.7% of CD4^+^ T cells respectively (Figure 1C). We also assessed Ki-67 expression (proliferation marker) in Treg within the 2 groups of patients, (Figure 1A, D-E). As shown in Figure 1D and E, the percentage of dividing Treg was similar in the tumors and peripheral blood for both tumor types.

### The phenotype of CD4^+^ CD25^+^ FoxP3^+^ T cells: TIL versus PBMC

**Figure 2:**
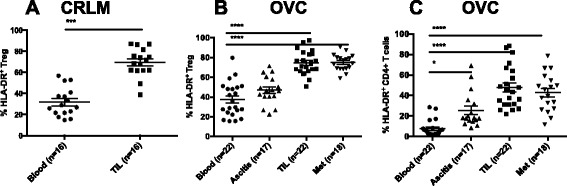
**HLA-DR expression on Treg and non-Treg CD4** ^**+**^ **T cells.** CD3^+^ CD4^+^ CD25^+^ FoxP3^+^ Treg cells were analyzed for HLA-DR expression in CRC samples **(A)** and OVC samples **(B)**. **C**. HLA-DR expression on CD4^+^ Foxp3^−^ non-Treg in OVC samples.

Since there are subtypes within Treg, that exhibit differing abilities to suppress T cell function, we determined whether the presence of these sup-phenotypes differed by location and tumor type. Because HLA-DR/MHC class II expression is a signature of activation for both Treg and effector CD4^+^ T cell populations we assessed its expression on TIL and PBL. As shown in Figure 2A and B, Treg present in the primary tumors and mets have increased HLA-DR expression compared to PBL for both CRC and OVC patients (69% and 70% compared to 31.7 and 37% respectively). We also found that the frequency of non-Treg/effector CD4^+^ T cells expressing HLA-DR is increased in the TIL compare to blood (Figure 2C). However, the mean fluorescense intensity (MFI) for HLA-DR was significantly greater in the Treg TIL compared to the conventional CD4^+^ TIL, 4225.8 and 4566.7 for primary tumor and omental metastases Treg compared to 2976.5 and 2639.8 for conventional CD4^+^ TIL in primary tumor and omental metastases (p = 0.0006 and <0.0001, respectively) (data not shown). The data suggest that the Treg within the tumor are more activated than the conventional CD4^+^ TIL within these progressively growing tumors.

**Figure 3:**
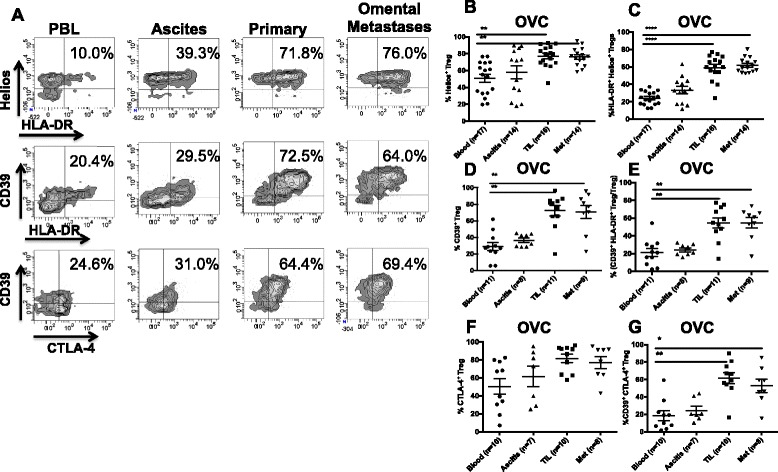
**Expression of markers characterizing more suppressive Treg.** Panel **A** shows the expression of Helios CD39 and CTLA-4 on CD4^+^ CD25^+^ Foxp3^+^ Treg from an OVC sample. **B**. Expression of Helios on Treg cells. **C**. Co-expression of Helios and HLA-DR on Treg (OVC n = 17). **D**. Percentage of CD39^+^ Treg, **E**. Percentage of CD39-HLA-DR Treg (OVC n = 11). **F**. CTLA-4 expression and **G**. CD39-CTLA-4 expression on Treg from OVC samples (n = 10).

Given that CRLM and OVC patients have a very different treatment and clinical history it was somewhat surprising that the increase in HLA-DR expression by Treg from tumors was consistent for both of these tumor types (Figure 2A-B). We further investigated whether other markers known to be expressed by activated Treg, were upregulated in the tumor microenvironment. We examined CD39, CTLA-4 and Helios all of which have been associated highly suppressive Treg activity. CD39 is an ectonucleoside triphosphate diphosphohydrolase, that hydrolases ATP and thus depletes ATP from the extracellular milieu and has been associated with highly suppressive Treg function [15,16]. Helios has been characterized as a Treg lineage marker and is a transcription factor that has also been associated with greater suppressive activity [17]. Fifteen OVC samples were analyzed for the expression of Helios and the frequency of Helios expressing Treg was significantly higher in the OVC primary tumors and omental metastases when compared to PBL (79.6, 76.2 versus 50.1%) (Figure 3A). Co-expression of HLA-DR and Helios was also higher on Treg from TIL (57.5%) and omental metastases (58.8%) compared to blood (23.6%) or ascites (34.7%) (Figure 3B-C). Eleven OVC samples were assessed for CD39 expression. Between 60-70% of Treg isolated from primary tumors and omental metastases were positive for CD39, while only 30% of the PBL Treg were positive for CD39 (Figure 3D-E). The MFI of CD39 was also significantly higher on Treg collected from TIL and Met compared to PBL (1842.5, 964.1 vs 441.9, with a p <0.001 and <0.05 respectively) (data not shown). When we assessed CD39 on the non-Treg CD4, 32.7% of TIL non-Treg CD4^+^ T cells expressed CD39 compared to 10.6% of PBL non-Treg CD4^+^ T cells (p <0.05) (data not shown). The CD39 MFI was greater on Treg compared to non-Treg CD4 T cell population. Co-expression of HLA-DR and CD39 was found on 50% of the Tregs within the primary tumors and Mets while only 20% of PBL Treg co-expressed these two markers (Figure 3E). Another inhibitory protein, CTLA-4 is expressed by both effector and regulatory T cells and it is known for suppressing T cell function [18]. There was no significant difference in percentage of Treg that expressed CTLA-4 in PBL, primary tumor and metastases Treg (Figure 3F). However, the co-expression of CTLA-4 and CD39 is significantly higher in the TIL (61.2%) compared to PBL (20.3%) (Figure 3G and Tables 4 and 5).

**Table 4:** **Percentage distribution of CD4** ^**+**^ **T cell phenotypes: summary of CD4** ^**+**^ **T cells phenotypes in CRLM patients**

**Mean% cell population (+/− SD)**	**PBL**	**TIL**
CD25+ Foxp3+ CD4+ T cells	8.1 (+/− 3.0)	10.2 (+/− 8.0)
Ki-67+ Treg cells	17.9 (+/− 7.6)	13.5 (+/− 9.4)
HLA-DR + Treg cells	31.7 (+/− 13.5)	69.4 (+/− 13.5)

**Table 5:** **Percentage distribution of CD4** ^**+**^ **T cell phenotypes: summary of CD4** ^**+**^ **T cells phenotypes in OVC patients**

**Mean% cell population (+/− SD)**	**PBL**	**Ascitis**	**TIL**	**Met**
CD25+ Foxp3+ CD4+ T cells	4.6 (+/− 3.4)	10.31 (+/− 4.4)	19.7 (+/−10.1)	19.8 (+/− 11.4)
Ki-67+ Treg cells	13.9 (+/− 8.2)	18.2 (+/− 12)	20.4 (+/−10.2)	22 (+/−11.3)
HLA-DR + Treg cells	37.4 (+/− 17.4)	47.0 (+/− 14.1)	74.6 (+/− 12.1)	75.1 (+/− 8.3)
HLA-DR + CD4+ T cells	7.6 (+/− 7.7)	25.5 (+/− 17.3)	48 (+/− 20.6)	42.7 (+/−18.2)
Helios + Treg cells	50.7 (+/− 19.8)	58.7 (+/− 27.1)	77.2 (+/− 12.6)	76.3 (+/− 11.0)
HLA-DR + Helios + Treg cells	24.1 (+/− 7.7)	35.1 (+/−15.9)	59.2 (+/− 14.2)	62 (+/− 8.7)
CD39+ Treg cells	29.1 (+/− 16.7)	43.1 (+/− 20.9)	72.2 (+/− 21.1)	67.5 (+/− 23.7)
HLA-DR + CD39+ Treg cells	21.1 (+/− 15)	28.8 (+/− 15)	54.4 (+/− 19.3)	53.4 (+/− 18.1)
CTLA-4 Treg cells	50.6 (+/− 27.8)	63.8 (+/− 28)	81.6 (+/− 14.9)	77.1 (+/− 20.2)
CD39+ CTLA-4+ Treg cells	18.5 (+/− 17.8)	27.2 (+/− 14.2)	61.7 (+/− 20.3)	53.6 (+/− 23)

### HLA-DR^+^ Treg isolated from tumors have greater suppressive function

**Figure 4:**
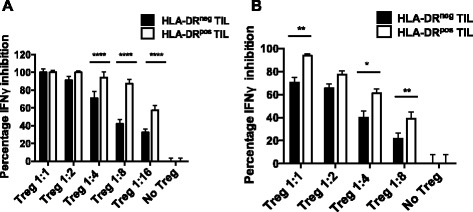
**HLA-DR + TIL Treg have a higher suppressive potency.** CD3^+^ CD4^+^ CD25^high^ and CD127^low^ Treg were sorted for HLA-DR+/−. Their suppressive function was measured in vitro by their capacity to suppress INFγ secretion by effector CD4^+^ T cells. Panel **A** and **B** represent 2 individual OVC patients.

We obtained sufficient surgical material from two OVC patients to compare the ability of HLA-DR^+^ and HLA-DR^−^ Treg to suppress IFNγ production from activated peripheral blood CD4 T cells. CD3 enriched T cells from tumors were stained with anti-CD4, anti-CD25, anti-CD127 and anti-HLA-DR and were gated on the CD25^hi^ CD127^lo^ CD4^+^ T cells, which identifies the Treg population in tumors [19,20]. These cells were then sorted into HLA-DR positive and negative subsets and their ability to inhibit T cell function was assessed. The HLA-DR^+^ Treg were 2–4 times more suppressive than HLA-DR^−^ Treg as measured by the inhibition of IFNγ secretion by activated CD4 T cells (Figure 4A-B and Additional file 2: Figure S2). These results are supportive of recent manuscripts [21,22] showing that the tumor microenvironment is greatly enriched for highly active Treg and this population most likely plays a role in dampening effector T cell responses within the tumor.

### Characterization of CD8^+^ TIL

**Figure 5:**
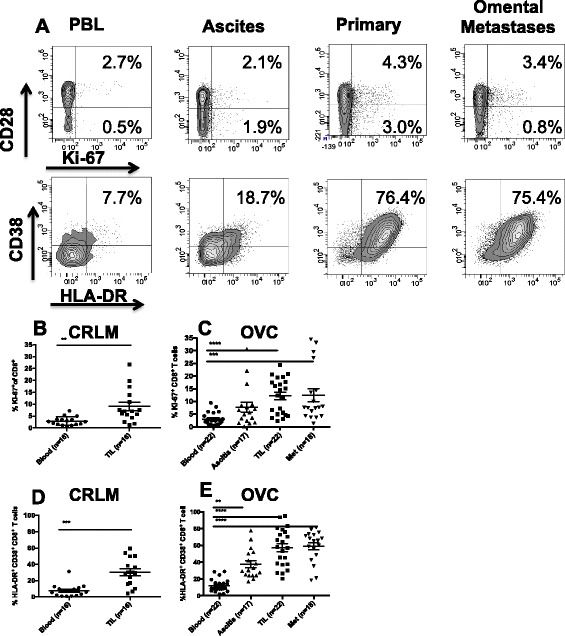
**CD8** ^**+**^ **T cells phenotype in CRC and OVC patients. A**. An example of flow cytometric analysis of CD3^+^ CD8^+^ T cells analyzed for CD28 and Ki-67, upper panels, and HLA-DR and CD38, lower panels. Percentage of Ki-67^+^ CD8 T cells in CRC samples (n = 16) **B**. and OVC samples (n = 22) **C**. Co-expression of CD38 and HLA-DR on CD8^+^ T cells in CRC samples **(D)** and OVC samples **(E)**.

CD8^+^ T cell immune infiltrates have been characterized in several different human tumor types [4]. In CRC, an increase in CD8^+^ infiltrates in the primary tumor has been shown to correlate with lack of metastases and decreased tumor progression [23,24]. The majority of these studies used IHC to assess cellular infiltrates [23,24] and very few studies describe complex CD8 phenotype and proliferative status of CD8 T cells isolated from tumors. In this study, we focused on co-expression of the membrane-bound activation markers CD38 and HLA-DR expressed on CD8^+^ T cells. Co-expression of these two proteins has been used to identify Ag-activated vaccine-specific CD8^+^ T cells in humans that had recently been inoculated with a yellow-fever vaccine [25]. Hence in the context of tumor infiltrating lymphocytes co-expression of these to markers may identify recently activated tumor-antigen specific T cells. The percentage of CD38^+^/HLA-DR^+^ CD8^+^ T cells in the peripheral blood of CRLM and OVC patients was low, between 8-10%. In both CRLM and OVC the percentage of CD38^+^/HLA-DR^+^ CD8^+^ T cells is increased five-fold and four-fold in TIL compared to PBL in ovarian cancer and CRC patients, respectively (Figure 5D-E). We also assessed Ki-67 expression to determine the proportion of dividing CD8^+^ T cells in both tumor types. The frequency of dividing PBL CD8^+^ T cells in both patient groups is approximately 3% (Figure 5A-C). However, the frequency of Ki-67^+^ CD8^+^ T is significantly higher in the tumors from CRLM patients, 9.1% and in the OVC primary tumors and omental metastases, 12.3% and 12.5% respectively, compared to PBL.

### Cytokine mRNA levels in CD4 T Cells from TIL versus PBL

**Figure 6:**
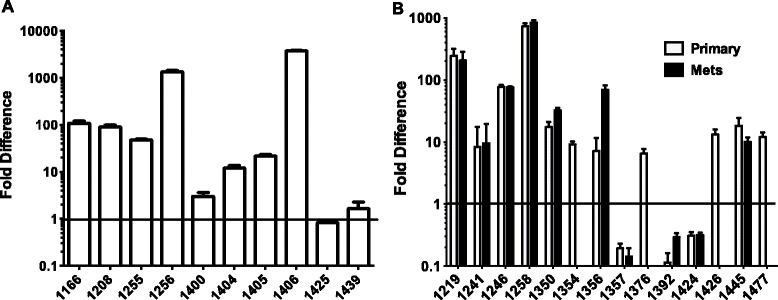
**Expression of IL-6 by qPCR in PBL and TIL.** CD4^+^ T cells were enriched from paired blood and tumor specimens and qPCR was performed on the enriched cell populations as described in Methods. The fold difference in IL-6 message in the TIL was normalized to the paired PBL in CRC samples **(A)** and OC samples **(B)**.

CD4^+^ T cells were enriched from both PBL and TIL as described in Methods. The purity of CD4^+^ T cells was between 85-95%. Cytokine mRNA was quantitated by PCR to investigate whether there were any differences in cytokine RNA obtained from blood versus tumor CD4^+^ T cells. No differences were observed for IL-2, INFγ, TGFβ, IL-3, IL-4 or IL-10 and very low levels of IL-17A and IL-21 mRNA were detected in both PBL and TILs. However, IL-6 mRNA was detected at high levels from CD4^+^ TIL when compared to PBL of the same patients (Figure 6). Quantitative PCR assessment for IL-6 in 14 OVC and 10 CRC patient samples showed significant increases in IL-6 mRNA production by CD4^+^ T cells when TIL were compared to PBL. In 7 out of 14 OVC patients samples, there was an increase in IL-6 mRNA both within the primary tumors and metastases when compared to blood, 4/14 revealed an increase in the metastases but not the tumor and for 3/14 there was no difference when comparing the blood, tumor and metastases. The fold-increase varied from 5-fold up to over a 1000-fold increase and the average was 12-fold. In the 10 CRC samples analyzed, 8 out of 10 revealed increased levels of IL-6 mRNA in TIL compared to blood. Similar to the ovarian cancer samples, the fold-increases varied from 2 to over a 1000-fold (average increase was 120-fold). We also quantified IFN-γ message from all the samples shown in Figure 6. As opposed to IL-6 no difference for IFN-γ mRNA was observed when comparing CD4 TIL to PBL within the same patient (data not shown).

## Discussion

This work demonstrates the utility of using a 10 color flow-cytometry panel for analyzing TIL from freshly isolated tumor samples and shows that extensive information can be obtained using this approach. 10-color flow cytometry allows for a more detailed phenotypic characterization of T cell subsets when compared to standard IHC techniques, which have been used in previous TIL studies [26]. These analyses show several significant phenotypic differences in the composition of TIL when compared to PBL. Our results also suggest, that regardless of the tumor type (CRC and OVC), the site of the disease (primary or metastatic lesions), or treatment status, the composition of TIL was surprisingly similar in these progressively growing tumors. We will began exploring other human malignancies in the future to ascertain whether this phenotypic pattern holds true across several different tumor types.

When comparing the TIL immune phenotype to PBL, we found several significant differences. OVC TIL contained a higher percentage of Treg compared to PBL (4.6% PBL compared to 19.7%), while there was no difference in Treg percentage in the CRC tumors compared to PBL. In both tumor types, Treg infiltrating the tumor were qualitatively different expressing higher levels of activation markers when compared to blood. These data show that proteins associated with a highly suppressive Treg phenotype [6,7,16,17] were more abundant in T cells that infiltrate tumors when compared to peripheral blood. These results also suggest that there is a selective accumulation of activated Treg within the tumor microenvironment regardless of the tumor type or location; primary or secondary site. These findings may lend credence to the hypothesis that the immune system fails to immunologically reject tumors because highly activated Treg are suppressing effector responses within the tumor microenvironment. Others have shown that the some of proteins studied within this manuscript mark Treg with a greater suppressive capacity [21,22]. This is an observation we confirmed by showing that sorted HLA-DR^+^ Treg were more suppressive when compared to HLA-DR^−^ Treg in two OVC patient tumor samples. Recent studies have shown that Treg isolated from CRLM have potent immune suppressive properties [27,28]. These studies did not analyze the phenotype of the Treg population, but they clearly demonstrated that Treg isolated from CRLM were more suppressive than Treg from PBL or tumor non-invaded tissue. We also found that infiltrating lymphocytes from both tumor types had a higher CD8/CD4 T cell ratio compared to PBL (see Table 1). Moreover, the CD8^+^ T cells within the tumor showed a greater propensity towards activation as measured by increased co-expression of CD38/HLA-DR. There was also an increased frequency of Ki-67^+^ CD8^+^ T cells in the TIL compared to PBL. While there is an increase in CD8 T cell activation and proliferation among patients’ TIL, it is clearly not sufficient to eradicate the tumor. There may be several explanations as to why these activated CD8 T cells are incapable of eradicating tumors. The CD8^+^ T cells within these tumors were found among suppressive cells including Treg (Tables 4 and 5), as well as MDSC [29] (not analyzed here), which could help to explain their failure to eradicate the tumor. These CD8 T cells could also express a host of exhaustive markers, such as PD-1 or Tim-3 (not assessed in this study), which could also limit their functional activity as shown by others [30,31]. Another possibility is that the cytokine milieu created by cells within the tumor microenvironment could have suppressive effects on T cell function. Hence, we also investigated levels of cytokine transcripts produced by CD4^+^ TIL isolated from progressively growing tumors. While we have found an increase in activated Treg, we also found that the CD4^+^ TIL express higher levels of IL-6 mRNA compared to CD4^+^ PBL. The increased expression of IL-6 mRNA observed within the tumor, if translated to protein, could also contribute to an immune-suppressed environment potentially through increased pSTAT3 upregulation within immune cells found in the tumor [32-34].

The two patient populations in this study had different clinical treatment histories. The CRLM patients typically had a preexisting history of cancer prior to the metastatic cancer resection, and in most cases had undergone previous resection of their primary tumor followed by chemotherapy (Table 1). In contrast, the patients with ovarian cancer typically had no treatment prior to their cytoreductive surgery. The tumor types analyzed also varied by stage of tumor development; liver metastatic tissue for CRC and primary tumors, omental metastases, and/or ascites for OVC patients. However, despite these differences, we observed many similarities in the TIL composition that were noted across different tumor types and sites of tumor growth.

Using a 10-color flow panel we provide a detailed analysis of the TIL composition from two different tumor types and found as expected that the TIL phenotype is much different than that of PBL. We can use these analyses as a phenotypic template of TIL isolated from progressively growing tumors and in the future compare these results to the phenotype of TIL isolated from patients treated with immunotherapy (e.g. anti-CTLA-4, anti-PD-1 and anti-OX40). Understanding how immunotherapies work by potentially changing the TIL composition and phenotype could lead to a more mechanistic approach to tumor immunotherapy, as most of the analyses in immunotherapy trials to date have analyzed PBL. This work also serve as a detailed platform for understanding the T cell phenotypes within progressively growing tumors and future studies will investigate the T cell phenotypes from other human malignancies in order determine similarities and differences to the results shown in this study. Ultimately, a greater understanding of the frequency and function of T cell infiltrates within progressively growing human tumors will allow us to better understand how to treat patients with immunotherapy to elicit T cell responses with greater clinical efficacy.

## Conclusion

The results presented in this study show that the composition of immune infiltrates isolated from patients harboring different tumor types is very similar. We found that both colorectal liver metastases and ovarian cancer have a greater percentage of activated T regulatory cells as well as a higher percentage of activated CD8^+^ T cells when compared to peripheral blood of the same patients. We also found that CD4 T cells isolated from both tumor types have an increase in mRNA for IL-6 compared to peripheral blood CD4 T cells. This manuscript provides a foundation for the activation profile of tumor infiltrating T cells in two different progressively growing tumor types and could be used as baseline when assessing immunologic changes in patients treated with immunotherapy.

## Methods

### Collection and isolation of lymphocytes from peripheral blood and ascites

Peripheral blood was collected in heparinized tubes just prior to or during surgery. Ascites cells were centrifuged and the pellet was resuspended in XVivo media (Lonza) and mononuclear leukocytes were separated from red blood cells and or tumor cells by Ficoll-Paque Plus (GE Healthcare, 17-1440-02) density gradient centrifugation for both ascites and peripheral blood. The leukocyte fraction was harvested, washed in PBS and counted prior to being stained or enriched.

### Isolation of tumor-infiltrating lymphocytes

Specimens were obtained at the time of surgery. Single cell suspension were obtained under sterile conditions in PBS, solid tumors were cut into 1-3 mm^3^ pieces and digested at room temperature for 1 hour on a magnetic stirring apparatus in a solution containing DNAse (Roche, 4536282001), collagenase (Sigma, C5138), halyuronidase (Sigma, H-6254) as well as human albumin (CSL Behring, 0053-7680-32) in RPMI (Lonza, 12-702Q). Enzymatically dissociated tumor was filtered through a 70 μm filter. Filtered samples were then diluted 1:2 with RPMI and ficolled as described above to obtain a leukocyte-enriched fraction. Cells were then washed three times in PBS (1^st^ wash @ 1,000 rpm for 10 minutes to remove debris, then 2x @1,250 for 5 minutes) and counted. Leukocytes were resuspended at 10 × 10^6^ cells/ml in PBS prior to staining or enrichment.

### Flow cytometry analysis

Single cell suspensions of leukocytes from blood, ascites, tumor or omental metastases were stained with the following monoclonal antibodies: anti-CD3 APC-H7 (BD Pharmingen, 560176), anti-CD4 eFlour 450 (eBioscience, 48-0048-42), anti-CD8 Pacific Orange (Invitrogen, MHCD0830), anti-CD25 APC (BD Pharmingen, 555434), or anti-CTLA-4 APC (BD Pharmingen, 555855), anti-CD28 PerCpCy5.5 (eBioscience, 45-0289-42), anti-CD38 PE-TR (Invitrogen, MHCD3817), anti-HLA-DR PE-Cy7 (BD Biosciences, 335795), anti-FoxP3 PE (eBioscience, 12-4777-42), anti-ki67 FITC (BD Pharmingen, 556026) or CD39 FITC (BD Pharmingen, 561444) or anti-Helios (Biolegend, 137214). Cells were stained in FACs buffer (1%FBS in PBS with 0.01%NaN_3_) and fixed according to the ebioscience FoxP3 Fix-Perm kit protocol (eBioscience, 00-5521-00). All samples were run on a BD LSRII Flow cytometer and analysed by FACSDiva BD. Briefly, for every single flow cytometric antibody, we have used Fluorescent Minus One (FMO), to discriminate between positive and negative cells [35].

### Treg sort

Lymphocytes from ascites, prepared as described above, were stained with anti-CD3, anti-CD4, anti-CD25, anti CD127 and anti-HLA-DR and sorted for T effector cells (CD3^+^, CD4^+^, CD25^low^ and CD127^high^) and Treg: CD3^+^, CD4^+^, CD25^high^, CD127^low^ and HLA-DR^+^ and CD3^+^, CD4^+^, CD25^high^, CD127^low^ and HLA-DR^−^. Cells were sorted on an Aria sorter (BD).

### Treg suppressive assay

Effector (30–50,000/well) and Treg were plated at ratio described in Figure 4, and stimulated with soluble anti-CD3 (1 μg/ml) and CD28 (2 μg/ml) as previously described [36]. Supernatents were harvested at Day 3 and 5 and IFNγ was measured by ELISA (eBioscience).

### CD4 enrichment

Enrichment of CD4^+^ T cells from blood and tumor samples was achieved using the EasySep Human CD4 Negative Enrichment kit (StemCell Technologies, 19052). The CD4 population was further purified, using the EasySep Human CD4 Positive Selection kit (StemCell Technologies, 18052).

### qPCR

The RNA from CD4^+^ lymphocyte populations was isolated using the QIAshredder (QIAGEN, 79654) and RNeasy Mini kits (QIAGEN, 74104). The isolated RNA was treated with Turbo DNase (Ambion, AM1907) and quantified by optical density. 1 μg of treated RNA was used to prepare cDNA using the RevertAid First Strand cDNA Synthesis kit (Fermentas, K1621). Samples were then analyzed by qPCR on the Applied Biosystems Step One Plus using 250 ng of cDNA per reaction with reactions set up in triplicate. The reactions utilized the Maxima Probe/ROX qPCR Master Mix (Fermentas, K0232). Samples were analyzed using FAM/TAMRA probes for IL-6 expression (Invitrogen, Hs00985639_m1), IL-17 expression (Invitrogen, Hs00174383_m1) and IFNγ expression (Invitrogen, Hs00989291_m1). GAPDH was used as the endogenous mRNA control (Invitrogen, Hs02758991_g1). cDNA from normal donor PBMCs stimulated with PMA/ionomycin for 11 hours was used as the calibrator sample. The results were then analyzed using the Comparative Ct Method of relative quantification.

## Additional files

Additional file 1: Figure S1 Flow cytometry gating strategy. Tumor samples were processed as described in Methods. Single cell suspensions were analyzed by flow cytometry. FSC and SSC were used to determine the lymphocyte population. CD3^+^ T cells were subdivided into CD4^+^ and CD8^+^ T cells. CD4^+^T cells were analyzed for the expression of CD25 and FoxP3 (Treg). CD39, HLA-DR, CTLA-4 and Helios were used to characterize highly suppressive Treg. Both non-Treg and Treg were assessed for proliferation by using Ki-67. Expression of CD38 and HLA-DR on CD8^+^ T cells was used to characterize recently activated CD8^+^ T cells and Ki-67 identified proliferating CD8^+^ T cells. Additional file 2: Figure S2 HLA-DR^+^ TIL Treg have a higher potency to suppress INFγ secretion. CD3^+^, CD4^+^, CD25^high^ and CD127^low^ Treg were sorted for HLA-DR^+^/_−_. They were co-cultured in triplicate with CD4^+^ CD25^−^ T cells at different cell ratio. INFγ secretion was measured in the supernatant by ELISA. Panel A and Panel B represent 2 individual patients.
