# Supplementary material for: Detailed characterization of tumor infiltrating lymphocytes in two distinct human solid malignancies show phenotypic similarities
Source: J Immunother Cancer. 2014 Nov 18;2:38. doi: 10.1186/s40425-014-0038-9 (PMC4247679; doi:10.1186/s40425-014-0038-9)
Supplement: Additional file 1: Figure S1 — Flow cytometry gating strategy. Tumor samples were processed as described in Methods. Single cell suspensions were analyzed by flow cytometry. FSC and SSC were used to determine the lymphocyte population. CD3+ T cells were subdivided into CD4+ and CD8+ T cells. CD4+T cells were analyzed for the expression of CD25 and FoxP3 (Treg). CD39, HLA-DR, CTLA-4 and Helios were used to characterize highly suppressive Treg. Both non-Treg and Treg were assessed for proliferation by using Ki-67. Expression of CD38 and HLA-DR on CD8+ T cells was used to characterize recently activated CD8+ T cells and Ki-67 identified proliferating CD8+ T cells. [file 40425_2014_38_MOESM1_ESM.pdf]

Supplemental Figure 1

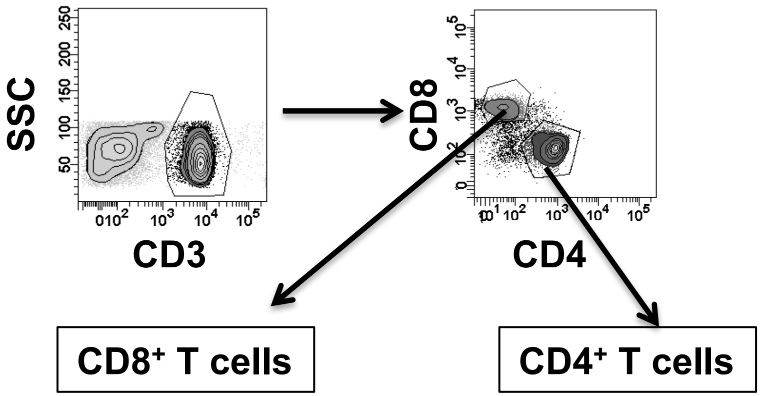

- CD38/HLA-DR: activation
- Ki-67: Proliferation

- CD25/Foxp3: Treg
- HLA-DR, CD39, CTLA-4: Treg phenotype
- Ki-67: Proliferation
