# Supplementary figures and images for: Detailed characterization of tumor infiltrating lymphocytes in two distinct human solid malignancies show phenotypic similarities
Source: J Immunother Cancer. 2014 Nov 18;2:38. doi: 10.1186/s40425-014-0038-9 (PMC4247679; doi:10.1186/s40425-014-0038-9)

Figure 2 Supplemental

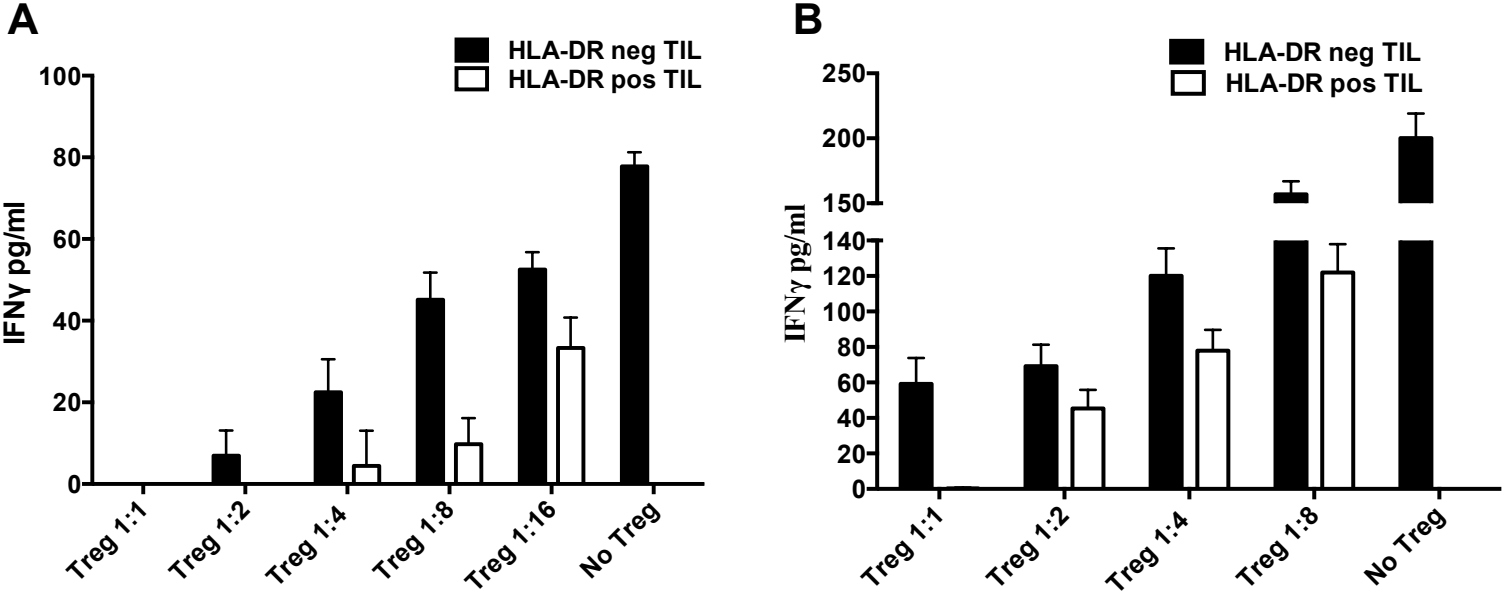

Supplement: Additional file 2: Figure S2 — HLA-DR+ TIL Treg have a higher potency to suppress INFγ secretion. CD3+, CD4+, CD25high and CD127low Treg were sorted for HLA-DR+/−. They were co-cultured in triplicate with CD4+ CD25− T cells at different cell ratio. INFγ secretion was measured in the supernatant by ELISA. Panel A and Panel B represent 2 individual patients. [file 40425_2014_38_MOESM2_ESM.pdf]
